# Water-absorbent beads-induced ileal obstruction in children: a retrospective case series and translational rat model study

**DOI:** 10.3389/fmed.2026.1980960

**Published:** 2026-10-27

**Authors:** Pengchong Dong, Mei Wu, Shasha Gong, Xuemei Fan, Qiang Zhao, Jianmei Yue, Baiyan Chen, Lixiang Wang, Jingjing Chen, Zhihui Teng, Zongyuan Wu, Chao Liu, Ying Xie, Weiguang Lian, Jingwei Xu, Libo Su

**Affiliations:** 1 Department of Laboratory Animals, Hebei Medical University, Shijia, Zhuang, China; 2 Department of Pediatric General Surgery, Hebei Children’s Hospital, Shijia, Zhuang, China; 3 Hebei Key Laboratory of Animal Science, Shijia, Zhuang, China

**Keywords:** gut microbiota, ileal obstruction, mucosal barrier, non-surgical animal model, pediatric foreign body, water-absorbent beads

## Abstract

**Background:**

Water-absorbent beads (WAB) may expand after ingestion and cause small-bowel obstruction in young children, but the associated intestinal injury and microbiota changes remain poorly defined. We combined pediatric case analysis with an experimental model to characterize WAB-induced ileal obstruction.

**Methods:**

Ten children who underwent surgery for WAB-induced ileal obstruction were retrospectively reviewed. A non-surgical rat model was established by intragastric administration of WAB. In total, 10 rats were assigned into two groups: WAB-obstruction group (*n* = 5) and sham control group (*n* = 5). Tissue assessment was performed at 48 h after gavage. Clinical features, gross and histologic injury, inflammatory and barrier-related gene expression, and 16S rRNA-based microbiota changes were assessed in the ileum, cecum, and colon.

**Results:**

In children, WAB were consistently impacted near the terminal ileum. Common presenting symptoms included vomiting and abdominal distension; complications such as perforation occurred only in a subset of these paediatric patients. In rats, 2–2.5 mm beads reproducibly induced ileal obstruction with weight loss, increased pain scores, ileal dilatation. Injury was segment-specific, with severe ileal mucosal damage, mild cecal changes, and relative colonic preservation. Ileal obstruction increased inflammatory cytokine expression, reduced *Muc2* and *Tjp1* expression, and was associated with enrichment of Pseudomonadota, Klebsiella, Proteus, and Escherichia-Shigella together with depletion of Lactobacillus and Akkermansia.

**Conclusions:**

WAB ingestion causes clinically important ileal obstruction in young children. This non-surgical rat model reproduces the composite pathological state of WAB-induced ileal obstruction frequently complicated by distant secondary intussusception, but it is not fully equivalent to uncomplicated paediatric WAB-induced ileal obstruction, and generalisation to all human WAB-obstruction cases should be exercised with caution. Our findings suggest that WAB-induced injury involves segment-specific mucosal damage, altered expression of barrier-related genes, inflammation, and microbiota dysbiosis.

## Background

Small-bowel obstruction is an important pediatric abdominal emergency that may progress rapidly and cause serious complications. Injuries associated with water-absorbent beads (WAB) have attracted increasing clinical attention in recent years. Their small size, bright colors, and availability create a risk of accidental ingestion among infants and toddlers (1, 2). Once hydrated, these beads can expand markedly within the digestive tract and remain radiolucent on conventional radiography. Recent case series and emergency epidemiological studies indicate that WAB ingestion primarily affects young children and may cause vomiting, fever, abdominal distension, intestinal obstruction, perforation, and retained beads after surgery, with some patients requiring endoscopic or surgical intervention (3, 4).

Unlike rigid foreign bodies, WAB are soft, deformable, and capable of substantial expansion after hydration. These properties increase the likelihood of impaction at anatomically narrow sites, particularly the distal ileum and ileocecal junction. At the impaction site, mechanical compression is accompanied by intestinal dilatation, local ischemia, mucosal barrier disruption and inflammatory changes. Associated complications reported in children include perforation, diffuse peritonitis and sepsis; intussusception has also been described as a rare associated event in isolated case reports (5, 6). Diagnosis is often delayed because the ingestion history is unclear and routine radiography may not detect the beads (6).

Current evidence on WAB is largely derived from case reports, retrospective case series, emergency database analyses, and *in vitro* expansion experiments (7). These studies have clarified the vulnerability of young children, the diagnostic limitations of radiography, and the need for endoscopic or surgical management in selected cases (8). However, the *in vivo* Pseudomonadota mechanisms of mucosal injury, inflammatory activation, and microbiota remodeling after WAB-induced obstruction remain poorly defined. Conventional animal models of intestinal obstruction usually depend on intestinal ligation, circumferential constriction, or torsion. Although these approaches produce stable obstruction, they also introduce incision trauma, intestinal manipulation, local ischemia, and surgery-related inflammation. These confounders can independently alter the mucosal barrier and gut microbiota (9), limiting their ability to model the ingestion-related obstruction caused by expandable foreign bodies.

The gut microbiota is central to mucosal integrity, immune homeostasis, and metabolic function (10). Mechanical obstruction can induce intestinal stasis, dysmotility, local hypoxia, and microbial restructuring, thereby aggravating inflammation and barrier injury (11). Studies of small intestinal obstruction have demonstrated associations between obstruction and reduced microbial diversity, enrichment of potential pathogenic taxa such as Pseudomonadota and Escherichia-Shigella, and predicted impaired metabolic function. Tight-junction regulation, SCFAs, and microbiota-mediated colonization resistance are also key determinants of epithelial barrier function and mucosal immune balance (12). Nevertheless, segment-specific pathological injury, barrier responses, and microbial remodeling after WAB-induced ileal obstruction remain insufficiently characterized.

Based on clinical features observed in our retrospective pediatric analysis of WAB-induced ileal obstruction, we established a non-surgical rat model via oral gavage to minimize surgical interference with intestinal mucosa and gut microbiota. In this animal experiment, we integrated histopathology, inflammation- and barrier-related gene expression, 16S rRNA gene sequencing, and predicted microbial functional profiles to evaluate segmental alterations in the ileum, cecum, and colon following WAB-induced obstruction (13, 14). These experimental findings provide an experimental framework for disease evaluation, clinical intervention, and mechanistic studies of pediatric WAB ingestion.

## Methods

### Ethics statement

The animal experimental protocol was approved by the Animal Care and Use Committee of Hebei Medical University (IACUC-Hebmu-2026028), and all animal experiments were performed in accordance with relevant institutional guidelines and the ARRIVE guidelines. Animals were monitored twice daily for body weight, activity, posture, grooming, and pain behaviours using the validated Roughan & Flecknell pain scoring system. Predefined humane endpoints were set as ≥20% body weight loss, persistent immobility, inability to access food and water, severe abdominal pain, or obvious signs of distress; animals reaching these endpoints were immediately euthanized. Isoflurane inhalation anesthesia was applied during bead intragastric administration. At the 48 h experimental endpoint, rats were euthanized under deep isoflurane anesthesia followed by cervical dislocation to confirm death, complying with AVMA guidelines.

All retrospective clinical analyses of pediatric patient data in this study were performed in full compliance with the ethical principles laid down in the Declaration of Helsinki. This retrospective clinical observational research was reviewed and approved by the Medical Research Ethics Committee of Hebei Children's Hospital (Ethics approval No. 2025001, simple review procedure). All clinical data of enrolled children were fully de-identified to protect patient privacy, and written informed consent was obtained from the legal guardians of all involved children prior to data collection. The ethics committee confirmed that all potential risks to participants were minimized in this research.

### Clinical data

Ten pediatric patients with accidental WAB ingestion were retrospectively identified from medical records of Hebei Children's Hospital between August 2021 and December 2025. Patient selection criteria included children with confirmed accidental WAB ingestion, surgically verified WAB-related ileal obstruction, and receiving surgical intervention. Patients with bowel obstruction caused by other etiologies were excluded. All 10 enrolled patients were confirmed to have ileal obstruction secondary to WAB ingestion.

Nine children underwent planned laparoscopic surgery under general anesthesia, mainly through an umbilical approach. One patient required conversion to laparotomy via a right mid-abdominal incision due to prolonged disease duration, difficult laparoscopic manipulation, and complex pathological findings. Longitudinal enterotomy for foreign-body removal, intestinal wall repair, or intestinal resection with anastomosis was performed according to injury severity. The abdominal cavity and the accessible intestinal segments were systematically explored to identify residual foreign bodies, detect missed perforation, and screen for concomitant gastrointestinal malformations.

### Animal models

Ten male Sprague-Dawley (SD) rats aged 6 weeks, with a body weight range of 120 ± 10 g, were purchased from Vital River Laboratory Animal Technology Co., Ltd. (Beijing, China). Animals were housed in a specific-pathogen-free facility at 22 ± 1 °C, 40%–60% relative humidity, and a 12-hour light/dark cycle, with free access to standard chow and water. A 7-day acclimatization period was performed before any experimental manipulation, with 2 rats per cage.

Prior to the main experiment, two independent preliminary screening experiments were conducted to select the appropriate diameter of WAB. In the first preliminary test, 15 SD rats were equally divided into 3 groups to test three bead diameters (1–1.5 mm, 2–2.5 mm, and 3–3.5 mm), with 5 rats per group. Two WAB beads were administered to each rat via gavage. All animals were euthanized after 24 h of observation, followed by gross anatomical examination. The results showed that beads of 1–1.5 mm passed out with feces without inducing intestinal obstruction; beads of 3–3.5 mm were retained in the stomach and failed to transit into the small intestine; only beads of 2–2.5 mm successfully reached the ileum within 24 h. In the second preliminary experiment, another 5 naive SD rats were used to verify whether the 2–2.5 mm beads could trigger ileal obstruction. Two WAB beads were gavaged in each rat, and the observation period lasted for 48 h. Continuous monitoring of general behavior and pain scoring was performed, and terminal gross anatomy was used to confirm the occurrence of ileal obstruction. This preliminary experiment verified that 2–2.5 mm WAB could stably establish the ileal obstruction model. All animals used in these two preliminary batches (total *n* = 20) were only used for gross phenotypic screening. Tissue and intestinal content samples from these preliminary rats were not subjected to histology, qPCR, or 16S rRNA microbiota sequencing.

For the main experiment, rats were randomly assigned to the WAB-induced obstruction group or PBS control group, with five rats per group. Randomization was performed using a simple random sampling method with random number table. The sample size *n* = 5 per group was determined by reference to previously published rat intestinal obstruction studies together with our preliminary experimental data, which showed that this sample size could detect differences in the main phenotypic and inflammatory indicators, while minimizing animal consumption following the 3R principle. Outcome assessors were blinded to group allocation during the assessment of pain score, body weight and gross pathological observation. Each rat in the obstruction group was administered 2 WAB beads. The beads were 2–2.5 mm in diameter before hydration, and were kept in dry state at the time of intragastric gavage. Beads were delivered with 0.1 mL PBS during gavage. The gavage procedure was performed using a flexible oral gavage tube. Successful obstruction was confirmed via gross anatomical observation at the 48 h experimental endpoint. The primary measurements including body weight, general condition and pain score were recorded every 12 h after gavage; intestinal tissues collected at the endpoint were used for histopathological examination and qPCR, while fecal samples were collected for 16S rRNA microbiota sequencing. The total number of experimental animals in this study was 30. All rats were euthanized by over-dose inhalation of 5% isoflurane in oxygen for at least 3 min to achieve deep anesthesia, followed by cervical dislocation as a secondary physical method to confirm permanent death at the experimental endpoint.

### Visceral pain behavioural assessment

Spontaneous abdominal pain behaviours were assessed at 48 h after WAB gavage, immediately before tissue harvest, using a 0–4 ordinal behavioural pain scale adapted from the behavioural ethogram. The original composite behaviour score counted the frequency of transient pain-related behaviours. Here, we adapted this validated behavioural repertoire into an ordinal severity scale, as this grading strategy has been widely adopted in rodent abdominal pain research to evaluate sustained visceral pain. Rats were kept undisturbed in their home cage. After a 2-min acclimatization period, behaviours were continuously recorded for 5 min under dim red light. Observed pain-associated behaviours included hunching/back arching, abdominal guarding, spontaneous locomotor activity and grooming behaviour. Scores ranged from 0 (no pain behaviour) to 4 (severe persistent abdominal pain). Two trained investigators performed all scoring, fully blinded to group allocation. The complete scoring criteria are shown in Supplementary Table S4 in the Supplementary Materials.

### Water-absorbent beads

WAB, cross-linked sodium polyacrylate, particle sizes: 1.0–1.5 mm, 2.0–2.5 mm, 3.0–3.5 mm, water absorption capacity 200–300 g/g, were purchased from Yiwu Jianchen Polymer Materials Co., Ltd. (Jinhua, China). The hydrated diameter of retrieved WAB was measured after the beads were recovered from the gastrointestinal tract at necropsy. The beads were gently rinsed with normal saline to remove surface debris before diameter measurement. All other reagents were of analytical grade and used as received.

### Routine blood examination

Peripheral blood was collected via cardiac puncture at the 48 h experimental endpoint under deep anesthesia with 4% isoflurane. Approximately 200 μL of whole blood was harvested and mixed with EDTA as the anticoagulant. Blood samples were analyzed immediately after collection. Peripheral blood samples were analyzed using an IDEXX ProCyte Dx automated veterinary hematology analyzer (IDEXX Laboratories, USA), which has species-specific validated algorithms for rat blood samples. The measured parameters included RBC, HGB, HCT, MCV, MCH, MCHC, RDW-CV, RDW-SD, PLT, MPV, PDW, PCT, WBC, Neu, Lym, Mon, Eos, and Bas. Both absolute cell counts and percentage values were reported for Neu, Lym, Mon, Eos, and Bas. The IDEXX ProCyte Dx analyzer directly generates true five-part leukocyte differential counts for these five leukocyte subsets automatically.

### Histological examination

After euthanasia, intestinal tissues were harvested. The 2 cm-long ileal segment was collected at the bead-induced obstructive site. A 2 cm-long sample was harvested from the cecal apex. For the colon, a 2 cm-long segment was obtained from the proximal one-third adjacent to the cecum. All tissue specimens were fixed in 4% paraformaldehyde for 24 h, embedded in paraffin, and cut into 4 μm thick cross-sections. Two cross-sections were prepared per intestinal segment per animal. Sections were stained with hematoxylin and eosin (H&E). Images were captured under an optical microscope at 200× magnification (20× objective lens). Three random non-overlapping fields of view were selected for each section. Histopathological scoring was independently performed by two assessors, who were blinded to the experimental group allocation. Scoring was performed according to the criteria listed in the supplementary table. During assessment, the assessors carefully distinguished true mucosal injury from artifacts generated during tissue fixation, embedding and sectioning.

### qPCR

Total RNA was extracted from ileal, cecal, and colonic tissues. The ileal sample was a 2 cm intestinal segment harvested proximal to the bead-induced obstructive site. The cecal sample was a 2 cm segment near the cecal apex collected after excision of tissue for HE staining. The colonic sample was a 2 cm segment obtained from the proximal one-third adjacent to the cecum. Approximately 50 mg of each tissue segment was used for RNA extraction. Freshly isolated tissues were immediately snap-frozen in liquid nitrogen and stored at −80 °C prior to RNA extraction. Total RNA was extracted using TRIzol reagent (Solarbio, R1100) following the manufacturer's instructions. RNA concentration and purity were determined by spectrophotometry (A260/A280 ratio). RNA integrity was verified by agarose gel electrophoresis. A total of 1 μg of total RNA was used for cDNA synthesis. DNase I treatment was performed before reverse transcription to eliminate genomic DNA contamination. RNA was reverse-transcribed into complementary DNA (cDNA) using the Thermo Scientific RevertAid First Strand cDNA Synthesis Kit (K1622). Gene expression quantification was performed on a QuantStudio 5 real-time PCR system. Gapdh was used as the reference gene. Relative mRNA expression was calculated using the 2^-*ΔΔ*Ct method. It should be noted that RT-qPCR detects mRNA transcript levels and does not directly represent protein abundance or intestinal barrier function.

### 16S rRNA gene sequencing and bioinformatic analysis

Ileal contents, cecal contents, and colonic fecal samples were collected from the corresponding anatomical sites in the Ob and Ctrl groups. 16S rRNA amplicon sequencing was performed by Lianchuan Biotechnology Co., Ltd. The workflow included DNA extraction, quality control, PCR amplification, product purification, library construction, NovaSeq sequencing, read merging and quality filtering, OTU clustering, taxonomic annotation, and alpha- and beta-diversity analyses. Venn diagrams were used to evaluate shared and unique OTUs. Principal coordinates analysis (PCoA) and principal component analysis (PCA) were used to assess community structure, and LEfSe was used to identify differential microbial biomarkers. PICRUSt2 was used to predict microbial functions from OTU sequences, and relative abundances of functional terms and metabolic pathways were assessed against the KO and KEGG databases.

### Statistical analysis

Statistical analyses were performed using GraphPad Prism, R software (version 3.4.4), and SPSS 26.0.Shapiro–Wilk test was used to assess normality, and Levene's test was used to evaluate homogeneity of variance. Only continuous data that passed normality testing and met the homogeneity of variance assumption, including body weight and qPCR gene expression data, were expressed as mea*n* ± standard deviation (SD) and compared by independent-samples t-test. Ordinal data, including histopathology scores and pain behavioural scores, were presented as median and interquartile range (IQR), and between-group comparisons were performed using the Mann–Whitney U test. A *P* value < 0.05 was considered statistically significant.

## Results

### WAB cause foreign body-induced digestive tract obstruction in children

Between August 2021 and December 2025, 10 pediatric patients underwent surgical treatment for ileal obstruction caused by accidental WAB ingestion at Hebei Children's Hospital. Among the 10 included patients, age ranged from 8 to 18 months. Disease duration, defined as the interval from symptom onset to surgical relief of obstruction, varied from 10 h to 6 days.

The distance of the bead from the ileocecal valve was measured proximally from the ileocecal valve along the intestinal lumen after surgical exposure. The beads were located 15–120 cm proximal to the ileocecal valve in these pediatric cases, indicating that the retained beads were distributed within the distal small intestine, with a subset positioned in the terminal ileum. The diameter of removed WAB ranged from 2.97 to 3.99 cm. Demographic characteristics, surgical procedures, and complications are summarized in Figure 1A.

**Figure 1:**
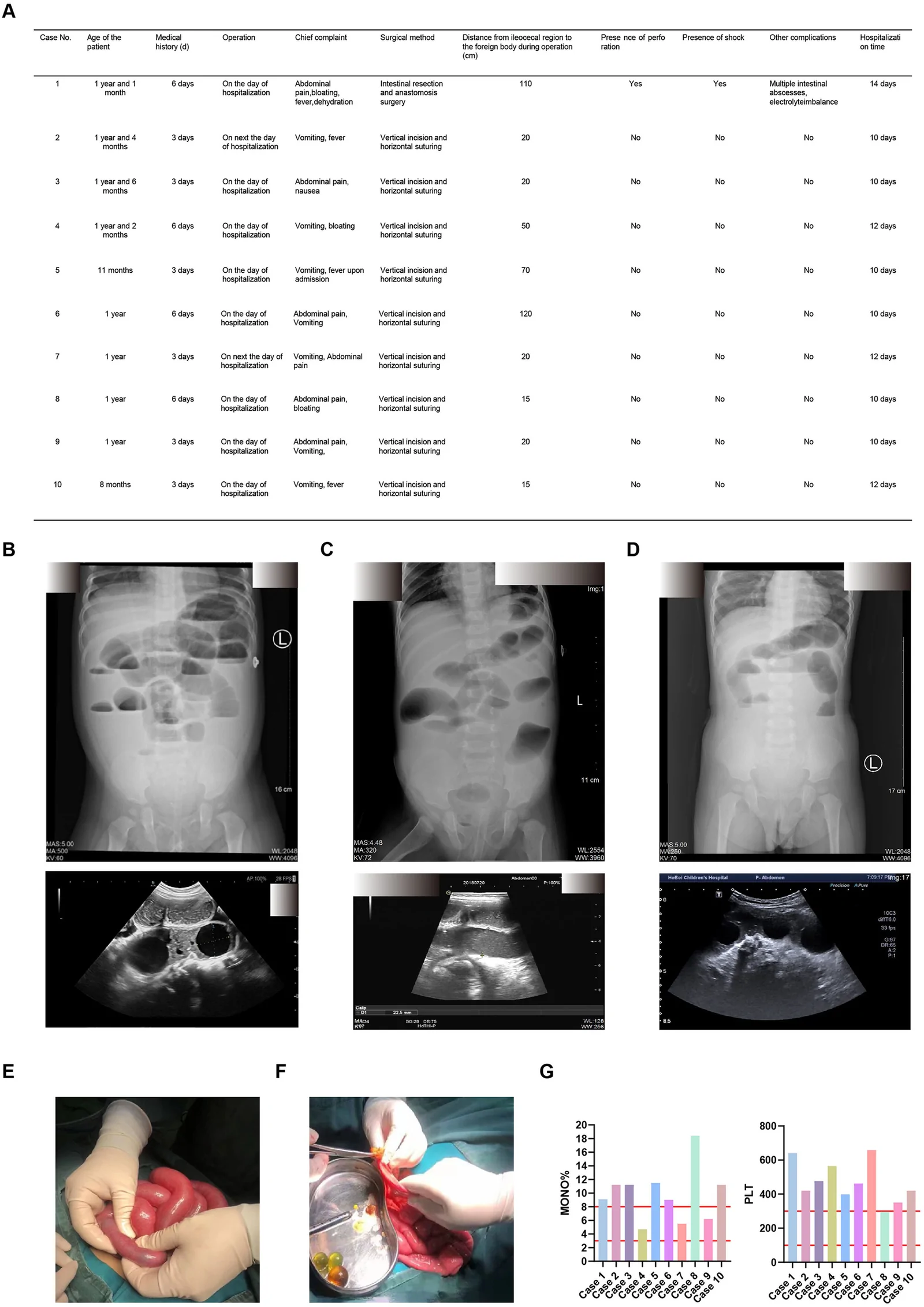
Water-absorbent beads ingestion causing intestinal foreign body obstruction in children. **(A)** Demographic characteristics, surgical approaches and complications of 10 enrolled patients. **(B–D)** Ultrasonographic manifestations of three representative clinical cases. **(E)** Intraoperative view showing round soft foreign body within intestinal lumen. **(F)** Maximum diameter measurement of extracted water-absorbent beads. **(G)** Monocyte percentage (MONO%) and platelet count (PLT) in ten pediatric patients with WAB-induced ileal obstruction. Data from all 10 patients with complete preoperative hematological datasets are presented in this panel. Red horizontal lines indicate the reference range of each indicator for this age group.

Three patients with complete preoperative imaging and surgical records were selected from this retrospective cohort to illustrate selected clinical presentations of WAB-induced ileal obstruction. Preoperative radiographs showed signs of intestinal obstruction, whereas ultrasonography suggested intraluminal foreign bodies, marked intestinal dilatation, and heterogeneous echoes (Figure 1B–D). During surgery, round and soft foreign bodies were identified within the small intestine (Figure 1E). The maximum diameter of the extracted beads was approximately 4 cm (Figure 1F), consistent with the imaging findings.

The main clinical manifestations were abdominal pain, abdominal distension, vomiting, and fever. Among the 10 enrolled pediatric patients with complete preoperative hematological records, preoperative hematological parameters from all 10 patients are graphically presented in Figure 1G. Routine blood tests showed increased PLT and MONO% in most patients, suggesting systemic inflammation or stress responses associated with WAB-induced obstruction.

### Passage test of different-sized WAB in the rat digestive tract

To simulate the clinical process of WAB-induced intestinal obstruction, gastrointestinal transit was first tested using WAB of different sizes in 6–7-week-old SD rats. WAB with an initial dry diameter of 1–1.5 mm (hydrated diameter: 7–8 mm) were spontaneously excreted and did not induce obstruction (Figure 2A). WAB with an initial diameter of 2–2.5 mm (hydrated diameter: 10–12 mm) reproducibly induced ileal obstruction and were selected for model establishment (Figure 2B). Larger WAB with an initial diameter of 3–3.5 mm (hydrated diameter: 15–18 mm) were prone to retention in the esophagus or stomach and did not reliably enter the small intestine through the cardia or pylorus (Figure 2C,D).

**Figure 2:**
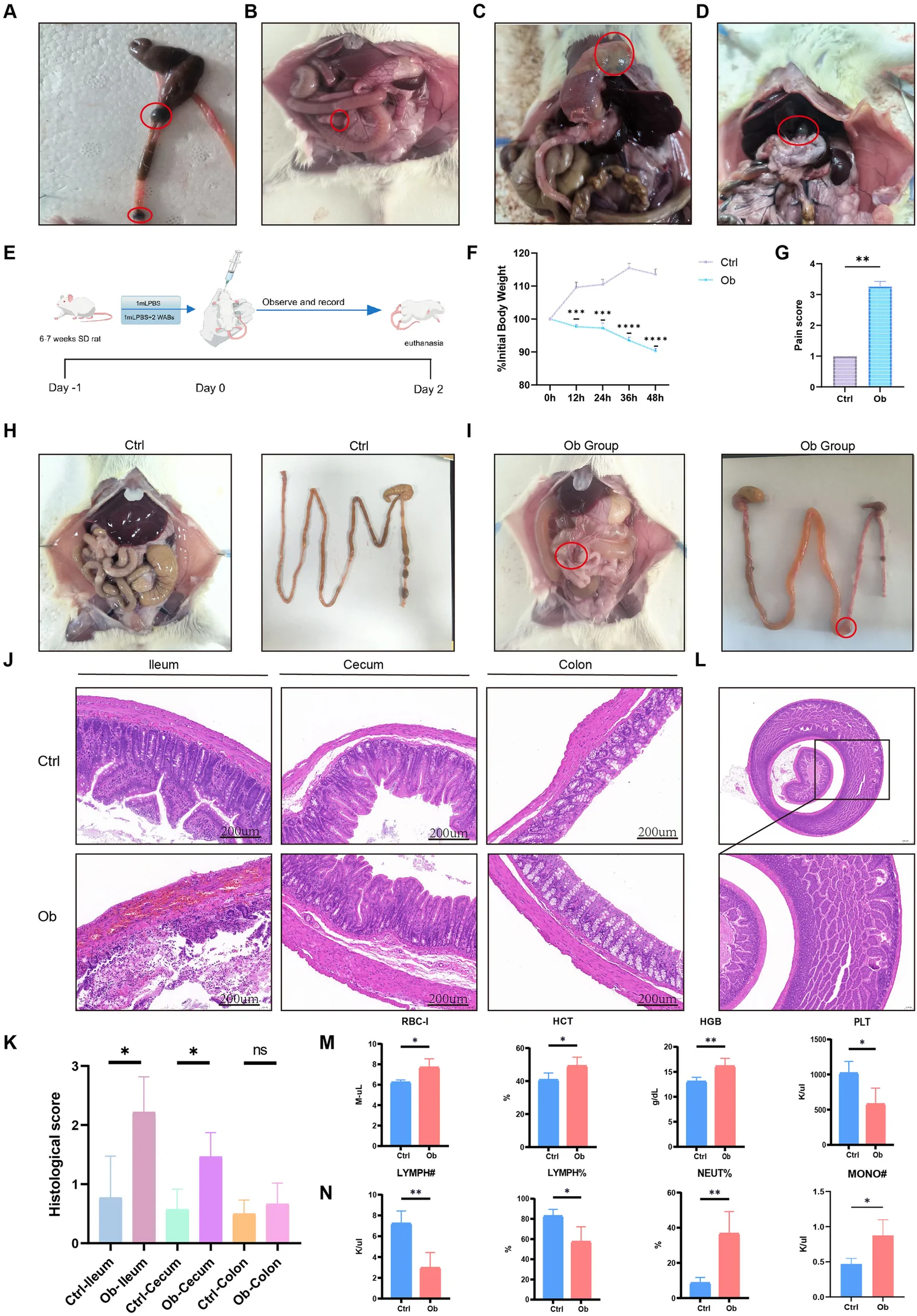
Establishment of non-surgical ileal obstruction rat model induced by water-absorbent beads**(A–D)** transit assessment of water-absorbent beads with different sizes in rat digestive tract.**(E)** Schematic diagram of intragastric administration and daily observation. Rats in obstruction group received two water-absorbent beads plus 1 mL PBS, while control group was given equal-volume PBS. All animals were monitored daily, weighed and euthanized at 48 h post treatment.**(F)** Line graph of body weight changes relative to baseline measured every 12 h.**(G)** Pain scores of the two groups at 48 h after gavage.**(H,I)** Gross intestinal morphological observation of control and obstruction groups.**(J,K)** Hematoxylin-eosin staining and pathological scoring of ileum, cecum and colon tissues.**(L)** Typical concentric ring structure observed in hematoxylin-eosin stained section of ileal intussusception.**(M,N)** Peripheral blood routine parameters of rats in two groups.

### WAB establish a non-surgical rat model of ileal obstruction

To mimic the natural progression of ileal obstruction after accidental WAB ingestion and to avoid traumatic interference from surgical manipulation, WAB with an initial diameter of 2–2.5 mm were paired with 6–7-week-old SD rats weighing 120–130 g to establish a non-surgical ileal obstruction model by oral gavage.

After 7 days of acclimatization, all rats were fasted for 12 h with free access to water. The Ob group received two WAB plus 1 mL PBS by oral gavage, whereas the Ctrl group received 1 mL PBS alone. Alertness and general behavior, feeding and drinking behavior, locomotor activity, and fecal characteristics were assessed at 12-hour intervals after gavage (Figure 2E). Relative body weight increased gradually in the Ctrl group but decreased continuously in the Ob group, with significant between-group differences (Figure 2F). At 48 h after gavage, pain scores were markedly higher in the Ob group than in the Ctrl group (*P* < 0.0001; Figure 2G). Rats in the Ob group progressively developed abdominal distension, hunched posture, and reduced defecation. Anatomical inspection showed ileal dilatation and fewer fecal pellets in the colon (Figure 2H,I).

H&E staining revealed distinct segment-specific pathological changes in the Ob group. The ileum showed the most severe injury, including disrupted mucosal architecture, villus atrophy, villus disruption and epithelial sloughing, epithelial necrosis, neutrophil infiltration in the lamina propria, submucosal congestion and edema, and focal erythrocyte extravasation. Histological scores were significantly higher than those in the Ctrl group (*P* < 0.05). The cecum showed mild pathological changes, including slightly shortened villi, sparse inflammatory-cell infiltration, and mild submucosal congestion, with significantly higher histological scores than controls (*P* < 0.05). In contrast, the colonic mucosal epithelium remained intact, with only a small number of lymphocytes in the lamina propria; the submucosa and muscular layer were preserved, and histological scores did not differ significantly between groups (*P* > 0.05; Figure 2J,K).

Notably, four of five rats in the Ob group developed ileal intussusception of varying severity. H&E staining showed a typical concentric ring-like structure (Figure 2L). The intussusceptum exhibited deformed villi, inflammatory-cell infiltration in the lamina propria, focal submucosal congestion and edema, and disorganization of the muscular layer. This intussusception represents a secondary incidental finding observed in this rat model.

Peripheral blood examination showed significantly higher RBC, HGB, HCT, and NEUT% in the Ob group than in the Ctrl group, whereas PLT, LYMPH#, and LYMPH% were markedly lower (Figure 2K,L). These changes indicate that WAB-induced ileal obstruction can trigger systemic inflammatory and stress responses.

### WAB induce local inflammatory responses at the obstruction site

To define segment-specific inflammatory responses after WAB-induced ileal obstruction, the relative mRNA expression of inflammatory cytokine-encoding genes was measured by qPCR in ileal, cecal, and colonic tissues.

In the ileum, the core obstructive site, *Il1b*, *Tnf*, *Il6*, and *Il10* mRNA levels were significantly higher in the Ob group than in the Ctrl group, indicating concurrent activation of pro-inflammatory responses and compensatory anti-inflammatory feedback (Figure 3A).

**Figure 3:**
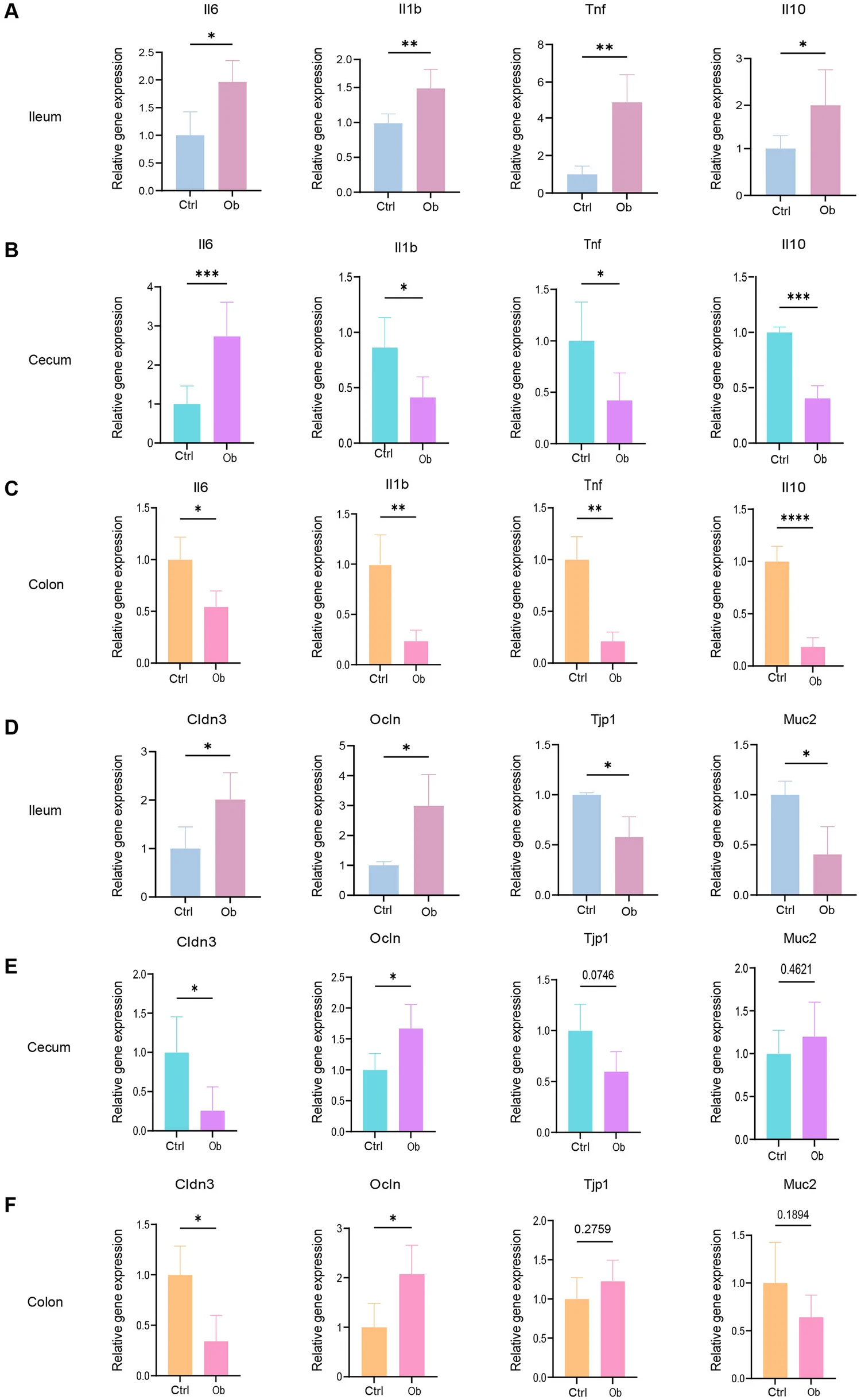
Water-absorbent beads induce inflammatory responses at the obstruction site**(A)** mRNA expression levels of *Il1b*, *tnf*, *il6* and *Il10* in the ileum of ob and ctrl groups.**(B)** mRNA expression levels of *Il1b*, *Tnf*, *Il6* and *Il10* in the cecum of Ob and Ctrl groups.**(C)** mRNA expression levels of *Il1b*, *Tnf*, *Il6* and *Il10* in the colon of Ob and Ctrl groups.**(D)** mRNA expression levels of *Cldn3*, *Ocln*, *Muc2* and *Tjp1* in the ileum of Ob and Ctrl groups.**(E)** mRNA expression levels of *Cldn3*, *Ocln*, *Muc2* and *Tjp1* in the cecum of Ob and Ctrl groups.**(F)** mRNA expression levels of *Cldn3*, *Ocln*, *Muc2* and *Tjp1* in the colon of Ob and Ctrl groups.

In the cecum, only *Il6* mRNA expression was increased in the Ob group, whereas *Il1b*, *Tnf*, and *Il10* expression was reduced (Figure 3B). In the colon, *Il1b*, *Tnf*, *Il6*, and *Il10* mRNA levels were all lower in the Ob group than in the Ctrl group (Figure 3C). These findings generally paralleled the segmental gradient of histopathological injury, although the mechanisms underlying cytokine downregulation in distal intestinal segments require further study.

### WAB-induced ileal obstruction alters mucosal barrier-related gene expression

Because mucosal barrier injury is a key pathological feature of mechanical intestinal obstruction, we measured genes associated with tight junctions and the mucus barrier in different intestinal segments.

In ileal tissues, *Cldn3* and *Ocln* mRNA expression was significantly higher in the Ob group than in the Ctrl group (*P* < 0.05), whereas *Muc2* and *Tjp1* expression was markedly reduced (Figure 3D). These findings suggest concurrent mucus-layer injury and intercellular-junction remodeling at the obstructive site, together with potential stress-induced or compensatory upregulation of selected tight-junction molecules.

In the cecum and colon, *Cldn3* mRNA expression was lower and *Ocln* mRNA expression was higher in the Ob group than in the Ctrl group. *Tjp1* and *Muc2* expression did not differ significantly between groups (*P* > 0.05; Figure 3F). The opposite trends in *Cldn3* and *Ocln* expression in distal intestinal segments may reflect reduced luminal contents, altered microbial composition, and adaptive alterations in intestinal barrier integrity.

### WAB-induced ileal obstruction alters gut microbiota diversity

Intestinal contents or fecal samples were collected from different intestinal segments. In the Ob group, 15–20 mg of ileal contents proximal to the obstruction site and cecal contents were collected, together with 1–2 fecal pellets from the colon. Samples of matched weight or quantity were collected from the corresponding sites in the Ctrl group. OTU analysis and Venn diagrams showed that ileal obstruction affected microbial composition in a segment-specific manner. The ileum had the lowest proportion of shared core OTUs and the clearest community separation between groups. The number of unique OTUs increased in the cecum of the Ob group, indicating microbial remodeling. The colon retained a relatively high proportion of shared OTUs and a more stable overall microbial composition. Rarefaction curves gradually plateaued with increasing sequencing depth, indicating sufficient sequencing coverage for subsequent analyses (Figure 4B).

**Figure 4:**
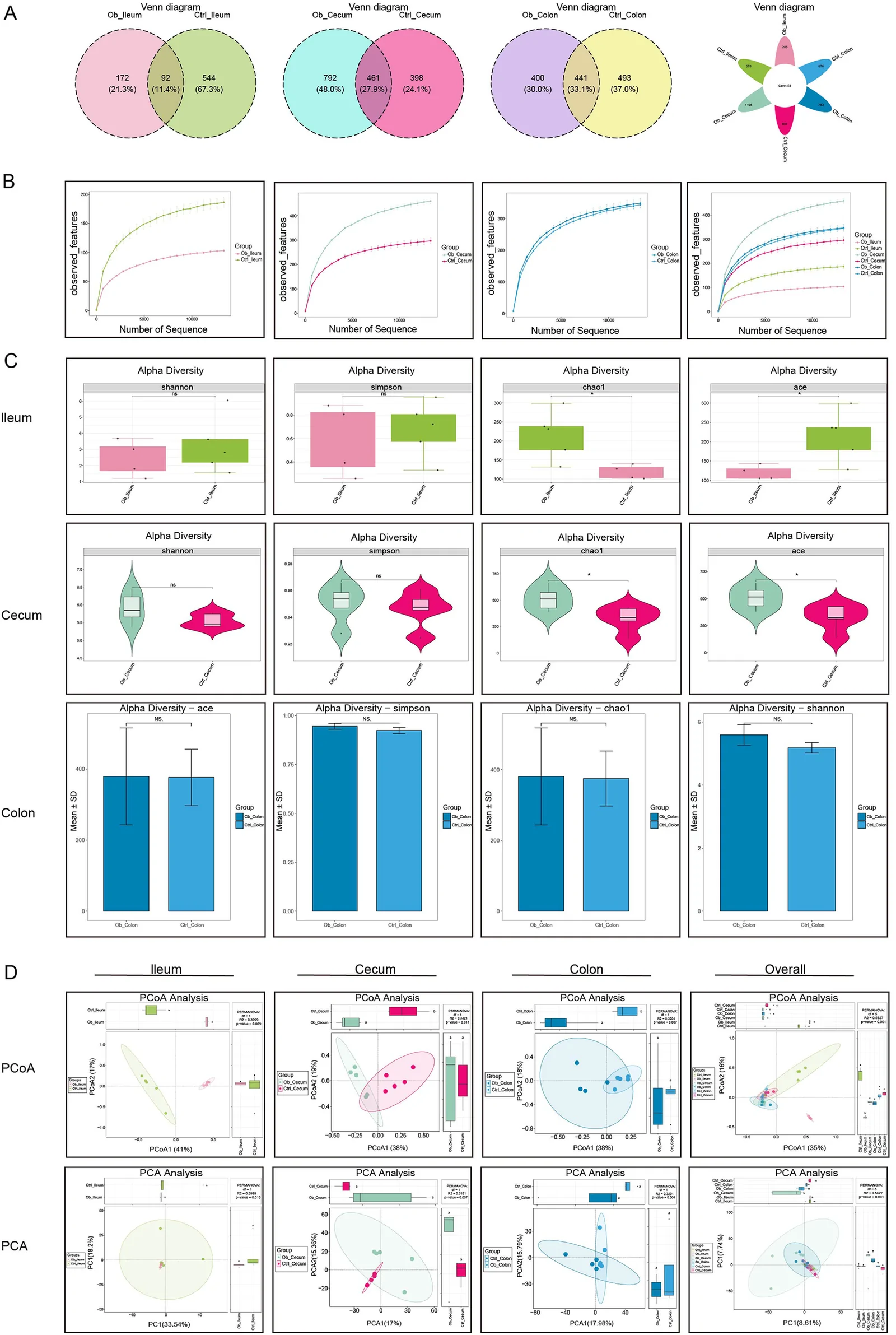
Ileal obstruction induced by water-absorbent beads alters intestinal microbiota diversity**(A)** venn diagrams based on OTU clustering of ileum, cecum, colon and overall microbiota.**(B)** Rarefaction curves of microbial communities in ileum, cecum, colon and total intestinal tract.**(C)** Alpha diversity analysis including Shannon, Simpson, Chao1 and ACE indices in ileal, cecal and colonic microbiota.**(D)** Beta diversity analysis via PCA and PCoA for microbiota from ileum, cecum, colon and whole intestine.

Alpha-diversity analysis showed significant differences in Chao1 and ACE indices between the Ob and Ctrl groups in the ileum and cecum (*P* < 0.05), indicating altered species richness. PCoA and PCA showed clear separation of ileal and cecal samples between groups, consistent with marked remodeling of microbial community structure. No significant differences in alpha or beta diversity were observed in the colon, suggesting that WAB-induced ileal obstruction had limited effects on distal colonic microbiota.

### WAB-induced ileal obstruction increases pseudomonadota abundance in the ileum

To characterize ileal microbiota composition, we analyzed taxa at the phylum and genus levels and identified differential biomarkers. At the phylum level, the relative abundance of Pseudomonadota was markedly higher in the Ob group than in the Ctrl group (*P* < 0.001), whereas Firmicutes abundance was significantly reduced (*P* < 0.001).

LEfSe analysis showed that the main differential biomarkers in the Ob group were Pseudomonadota, Escherichia-Shigella, Klebsiella, and Proteus, whereas the Ctrl group was characterized by Firmicutes, Actinobacteriota, and Lactobacillus (Figure 5E). These results suggest that WAB-induced obstruction shifts the ileal microbiota from a commensal state toward a potentially pro-inflammatory profile.

**Figure 5:**
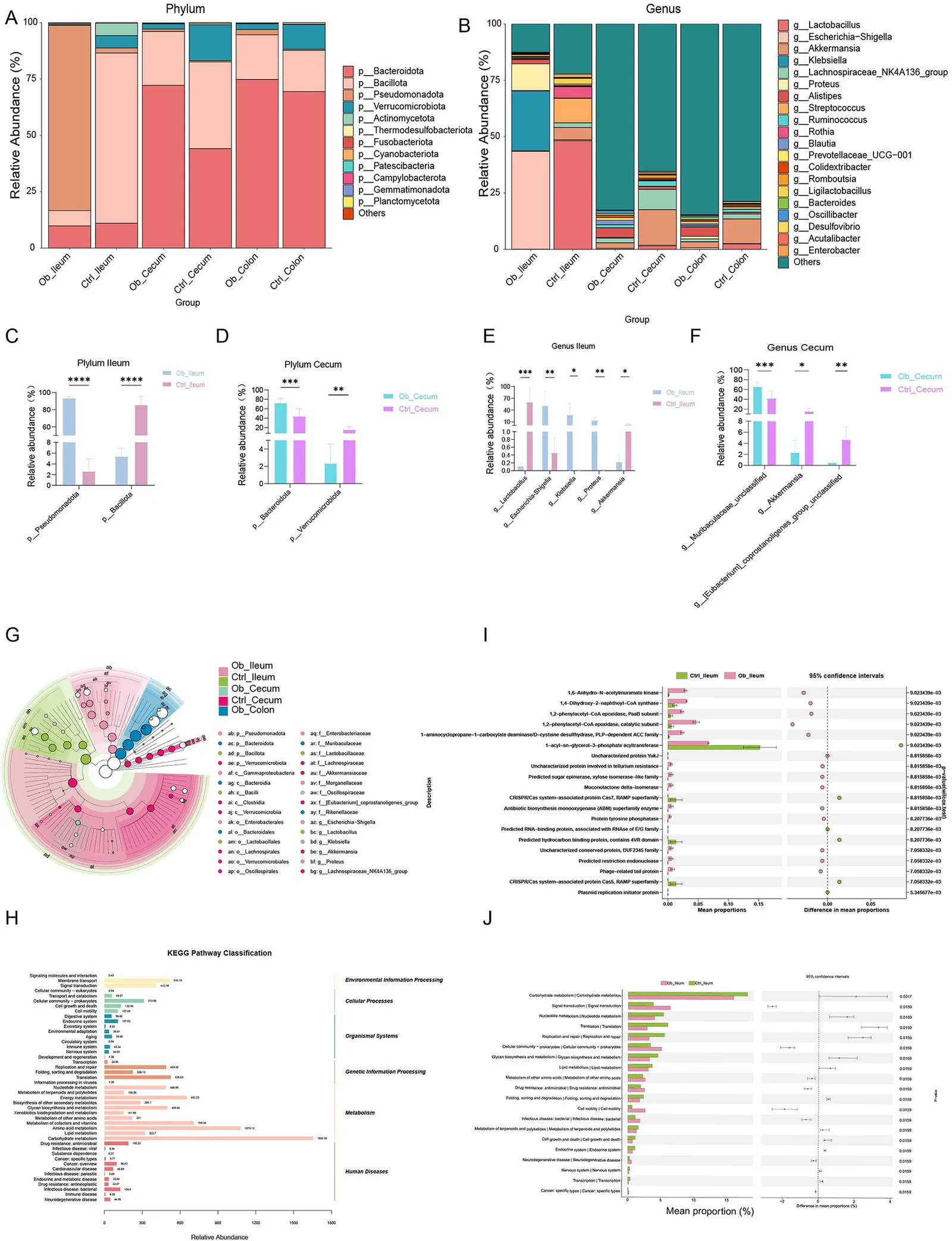
Increased abundance of pseudomonadota and functional prediction analysis following water-absorbent bead-induced ileal obstruction**(A)** histogram showing relative abundance of the top 12 phyla of intestinal microbiota.**(B)** Histogram showing relative abundance of the top 20 genera of intestinal microbiota.**(C,D)** Phylum-level microbial differences in ileum and cecum.**(E,F)** Genus-level microbial differences in ileum and cecum.**(G)** Cladogram with concentric circles radiating outward representing taxonomic ranks from phylum to genus in ileum, cecum and colon. Circle diameter corresponds to microbial relative abundance, and color denotes predominant taxa in each group.**(H)** Overall KEGG functional prediction of gut microbiota. Pathway classification was annotated using the KEGG database (map01100; https://www.kegg.jp/kegg/kegg1.html), with formal publication permission granted by Kanehisa Laboratories (Ref: 261577). **(I)** KEGG functional analysis focused on ileal microbiota. Functional annotation was performed against the KEGG database (map01100; https://www.kegg.jp/kegg/kegg1.html), with formal publication copyright permission (Ref: 261577) obtained from Kanehisa Laboratories. **(J)** Differential analysis at KO level. Functional annotation was performed against the KEGG database (map01100; https://www.kegg.jp/kegg/kegg1.html), with formal publication copyright approval (Ref: 261577) granted by Kanehisa Laboratories.

### WAB-induced ileal obstruction reshapes cecal microbiota composition but has limited effects on the colon

In the cecum, the Ob group showed significantly higher relative abundance of Bacteroidota (*P* < 0.001) and markedly lower relative abundance of Verrucomicrobiota (*P* < 0.01) than the Ctrl group. At the genus level, unclassified Muribaculaceae was enriched in the Ob group, whereas Akkermansia and the Eubacterium coprostanoligenes group were depleted (Figure 5B,D). LEfSe analysis identified Oscillospiraceae as the main differential biomarker in the Ob group, whereas Verrucomicrobiota, Akkermansia, Lachnospiraceae NK4A136 group, and Eubacterium coprostanoligenes group were characteristic taxa in the Ctrl group (Figure 5E).

In the colon, Bacteroidota was the dominant bacterial phylum in both groups. LEfSe analysis identified only Bacteroidota-centered differential biomarkers in the Ob group, indicating that WAB-induced ileal obstruction had a relatively limited impact on colonic microbial community structure (Figure 5E).

### Functional prediction analysis of ileal microbiota alterations

Because the ileum was the primary site of injury after WAB-induced obstruction, we assessed the potential functional consequences of ileal microbiota dysbiosis by integrating microbial composition, LEfSe biomarkers, KO enrichment, and KEGG pathway prediction.

KEGG functional prediction indicated that carbohydrate metabolism was a major microbial functional category in both groups (Figure 5F). In the ileum, carbohydrate metabolism accounted for the highest proportion of predicted pathways in both groups, but its relative proportion was slightly lower in the Ob group than in the Ctrl group (Figure 5G). This trend suggests that WAB-induced obstruction may alter the metabolic potential of the ileal microbiota, including pathways related to SCFA production, epithelial energy supply, barrier repair, and local immune homeostasis. Because SCFAs were not directly measured, this interpretation requires validation by targeted metabolomics or related assays.

KO-level differential analysis showed significant enrichment of functional terms including 1,6-anhydro-N-acetylmuramate kinase and 1,2-phenylacetyl-CoA epoxidase in the Ob group (*P* < 0.05). These terms are associated with bacterial cell-wall metabolism and secondary metabolism, which may reflect enhanced environmental adaptation of potentially pathogenic bacteria after obstruction. Collectively, these findings support a model in which WAB-induced obstruction promotes localized ileal injury through interactions among mechanical compression, barrier disruption, microbiota dysbiosis, and inflammatory amplification.

## Discussion

This study combined 10 pediatric cases of WAB-induced ileal obstruction with a non-surgical SD rat model established by intragastric gavage. We systematically analyzed the clinical features, histopathological alterations, mucosal barrier responses, and microbiota remodeling associated with obstruction caused by soft expandable foreign bodies. The main findings were that WAB caused typical ileal obstruction in young children. WAB with an initial diameter of 2–2.5 mm reproducibly induced non-surgical ileal obstruction in 6–7-week-old SD rats; tissue injury showed clear segment specificity, with severe mucosal destruction in the ileum, mild secondary changes in the cecum, and relative preservation of the colon; and obstruction remodeled the ileal and cecal microbiota, with enrichment of Pseudomonadota and potential pathogens and depletion of Lactobacillus and Akkermansia. The main contribution of this work is therefore not only to confirm that WAB can induce intestinal obstruction, but also to provide a reproducible and minimally invasive model that distinguishes local injury from distal adaptive responses.

The clinical findings are consistent with recent English-language literature. Emergency database studies have reported a sharp increase in pediatric emergency visits related to WAB from 2021 to 2022, with children younger than 5 years being the most vulnerable group and ingestion being a major injury mechanism (7). A recent single-center case series reported a median age of approximately 1 year and 4 months among children with superabsorbent polymer bead ingestion; common manifestations included vomiting, fever, and abdominal distension, and some patients developed obstruction requiring endoscopic or surgical intervention (5). Reports in the Journal of Pediatric Gastroenterology and Nutrition, Pediatrics, and BMC Pediatrics have emphasized the radiolucency, persistent expansion, frequent missed diagnosis, and potential severity of WAB/SAPB injuries (15). In the present cohort, we enrolled 10 pediatric patients undergoing surgery for WAB-induced obstruction. All retrieved beads (foreign bodies) were found 15–120 cm proximal to the ileocecal valve. Several patients had perforation or systemic inflammatory features. These data indicate that retained beads were distributed within the distal small intestine, with the ileocecal region representing an important anatomical zone for risk assessment (1).

The non-surgical model developed here has methodological value. Traditional intestinal obstruction models usually rely on ligation or torsion, but surgical trauma can independently alter intestinal perfusion, mucosal integrity, immune responses, and microbial composition. In contrast, our model used intragastric gavage without abdominal incision or direct intestinal manipulation, avoiding trauma, incision-derived ischaemia and surgical inflammation caused by laparotomy, bowel ligation or torsion. It should be emphasised that this model does not exclude pathological ischaemia and inflammation originating from disease-secondary complications such as spontaneous intussusception occurring downstream of WAB-induced intestinal distension. This model reflects the pathological process of foreign-body expansion and local incarceration after accidental WAB ingestion. The animals developed weight loss, increased pain-related behavior, ileal dilatation, and reduced fecal pellets, indicating that the model recapitulates major clinical phenotypes of WAB-induced ileal obstruction. Secondary ileal intussusception occurred as an incidental complication unique to this rat model. Particle-size testing further showed that obstruction depends not simply on the presence of a foreign body, but on the dynamic relationship among initial bead diameter, post-expansion volume, intestinal lumen diameter, and gastrointestinal transit. Compared with surgical partial small-bowel obstruction models, this model provides an alternative experimental platform for investigating early mucosal injury and microbiota changes induced by soft foreign-body incarceration (9). It should not, however, replace ligation, torsion, or ischemia/reperfusion models when the research question concerns vascular interruption, necrosis, or fibrotic repair.

Segment-specific intestinal injury was the central pathological feature of this study. WAB-induced obstruction did not produce homogeneous injury throughout the intestine; instead, lesions were centered on the ileum, the primary obstructive site. The ileum was directly exposed to foreign-body compression, intestinal dilatation, and luminal-content accumulation, resulting in villus destruction, epithelial necrosis, submucosal edema, and neutrophil infiltration (16). The cecum was not directly incarcerated but showed mild inflammatory stress, probably secondary to upstream obstruction, altered luminal contents, and bacterial translocation (17). The colon, which was more distant from the obstruction site, was less affected by disrupted transit and microbial perturbation and therefore maintained a relatively stable tissue structure and inflammatory status (12). These findings suggest that treatment and postoperative evaluation should focus on tissues proximal to the obstruction and around the ileocecal region rather than assuming uniform intestinal injury. They also highlight the importance of segment-specific sampling, because mixed fecal sequencing may dilute local microbial signals in focal obstructive disease.

Inflammatory cytokine and barrier-related gene changes further supported the segment-specific pattern. Concurrent upregulation of ileal *Il1b*, *Tnf*, *Il6*, and *Il10* mRNA suggests acute pro-inflammatory activation together with compensatory anti-inflammatory feedback (18). The downregulation of *Muc2* and *Tjp1* mRNA in the ileum indicates impairment of the mucus-layer-associated MUC2 and tight-junction protein ZO-1 (19), whereas increased *Cldn3* and *Ocln* mRNA expression may reflect stress-induced or reparative remodeling after mechanical traction and inflammatory stimulation. Tight-junction expression does not have a simple linear relationship with barrier integrity; expression level, subcellular localization, and phosphorylation state jointly determine paracellular permeability (2). Therefore, the mRNA results should be interpreted as evidence of barrier-remodeling signals rather than direct proof of preserved or impaired barrier function. Additional validation by immunofluorescence, western blotting, permeability assays, and protein-localization analysis is required.

The microbiota data provide insight into the spatial microecological effects of WAB-induced obstruction. Significant changes in microbial diversity and community structure were observed in the ileum and cecum, whereas the colon remained relatively stable, indicating spatially restricted microbial perturbation. In the ileum, Pseudomonadota, Klebsiella, Proteus, and Escherichia-Shigella were enriched, whereas Lactobacillus and Akkermansia (20) were depleted (21). This pattern is consistent with microbial signatures commonly seen in inflammatory or obstructive conditions, including pathobiont expansion, weakened colonization resistance, and loss of beneficial bacteria (22). Similar findings have been reported in partial small intestinal obstruction models, including reduced diversity, Pseudomonadota enrichment, reduced Akkermansia abundance, and impaired metabolic function.

Functional prediction suggested a relative reduction in carbohydrate metabolism in the ileal microbiota of the Ob group, accompanied by enrichment of KO terms related to cell-wall and secondary metabolism. All functional annotations were generated based on the KEGG global metabolic pathway database (map01100), and the official copyright permission (Reference No. 261577) from Kanehisa Laboratories was obtained for the publication of these KEGG-derived statistical charts in this manuscript (Kanehisa & Goto, 2000). Given the roles of SCFAs in epithelial energy supply, barrier stabilization, and immune regulation (23), these findings raise the hypothesis that WAB-induced obstruction may promote a cascade involving mechanical compression, alterations in intestinal barrier integrity, microbiota dysbiosis, and inflammatory amplification through loss of beneficial bacteria, reduced metabolic support, and expansion of potential pathogens. However, PICRUSt2 infers functional potential from 16S rRNA sequencing and cannot substitute for metagenomic, transcriptomic, or metabolomic evidence (24).

Interpretations involving SCFAs or virulence-related metabolism should therefore be regarded as mechanistic hypotheses rather than definitive causal conclusions.

The findings also have clinical implications. In young children with suspected WAB ingestion, risk of obstruction in the terminal ileum and ileocecal region should be assessed using ingestion history, symptom progression, and ultrasonography or computed tomography (CT), even when plain radiography is negative (8). During surgery, the accessible intestinal segments should be carefully explored, with attention to residual foreign bodies, intussusception, and perforation in the distal small intestine. These findings generate the hypothesis that postoperative management research may explore targeted strategies focused on ileal mucosal protection, inflammation control, infection surveillance, and microbiota restoration, rather than relying only on nonspecific intestinal support. Further studies are required to validate whether such strategies confer clinical benefits. From a public-health perspective, WAB are non-food high-risk foreign bodies, and stronger warning labels, household education, and age-restricted access are warranted (25).

This study has several limitations. First, the animal experiment had a small sample size and included only one observation time point (48 h), limiting assessment of disease onset, progression, and recovery. Second, inflammatory and barrier molecules were measured only at the mRNA level, without protein-expression, spatial-localization, or permeability validation. Third, microbial functions were inferred from 16S rRNA sequencing, and SCFAs, endotoxins, bile acids, and other key metabolites were not directly quantified. Fourth, intervention experiments using antibiotics, probiotics, fecal microbiota transplantation, or obstruction relief were not performed, so it remains unclear whether microbiota changes are drivers or consequences of intestinal injury. Fifth, the clinical cohort was small, and mechanistic validation using pediatric mucosal or fecal samples was unavailable. Sixth, reduced food intake and potential dehydration secondary to ileal obstruction may independently alter gut microbiota and intestinal physiology. Both groups received identical 12-h pre-gavage food-water deprivation; however, post-gavage spontaneous reduction in food and water consumption occurred only in the obstruction group. Since microbiota alteration is a secondary readout for model characterization rather than the primary endpoint of this study, a pair-fed control group was not included. Accordingly, we cannot fully disentangle microbiota changes directly caused by mechanical obstruction from those secondary to decreased food and water intake. Future studies focusing primarily on microbiota outcomes should incorporate pair-fed experimental groups to resolve these confounding effects. Seventh, 80% of animals in the obstruction group developed distant secondary ileal intussusception, whereas none of our 10 pediatric patients presented this complication. This marked inter-species discrepancy likely arises from differences in intestinal anatomy and gut motility between rats and human children. Although all histological and qPCR samples were collected from the bead-obstructed intestinal segment (avoiding tissue from the intussusception lesion itself), indirect systemic and inter-segment crosstalk originating from distant intussusception cannot be completely excluded. Given the small sample size of the main experiment (*n* = 5 per group), subgroup comparison between animals with- vs.-without intussusception was not statistically feasible. Therefore, we cannot definitively separate pathological signals caused by bead-mediated mechanical compression from indirect effects secondary to distant intussusception. Larger-scale animal studies are required in future work to compare uncomplicated WAB-obstruction versus obstruction complicated by secondary intussusception. Finally, SD rats were used to model WAB-induced obstruction in infants; differences in age, intestinal lumen size, diet, and baseline microbiota mean that the model is most appropriate for mechanistic exploration and hypothesis generation, with further clinical validation required.

Further work could combine protein-expression assays, immunofluorescence and intestinal permeability testing to comprehensively evaluate epithelial barrier integrity. Integration of metagenomic and metabolomic analyses would help quantify microbial functional potential and metabolite concentrations. Intervention experiments targeting selected obstruction-associated bacterial taxa or metabolites may help clarify their contribution to mucosal injury. Time-course experiments at 6, 24, 48, and 72 h and after obstruction relief should define the temporal sequence of tissue injury, inflammation, alterations in intestinal barrier integrity, and microbiota changes (26). Microbiota-based intervention experiments should test whether beneficial bacterial supplementation, targeted inhibition of Pseudomonadota, or metabolite supplementation can reduce mucosal injury (27).

## Conclusions

This study established a non-surgical rat model of ileal obstruction through intragastric administration of WAB. WAB-induced obstruction was associated with segment-specific mucosal injury, alterations in intestinal barrier integrity, inflammatory activation, and gut microbiota dysbiosis, with the most pronounced changes in the ileum. This model provides a preclinical platform for investigating intestinal obstruction associated with soft, expandable foreign bodies. Further studies are needed to determine the translational relevance of these findings to children after accidental WAB ingestion.

## Data Availability

The datasets presented in this study can be found in online repositories. The names of the repository/repositories and accession number(s) can be found in the article/Supplementary Material.
