# Supplementary material for: Water-absorbent beads-induced ileal obstruction in children: a retrospective case series and translational rat model study
Source: Front Med (Lausanne). 2026 Oct 27;13:1980960. doi: 10.3389/fmed.2026.1980960 (PMC13747860; doi:10.3389/fmed.2026.1980960)
Supplement: Supplementary file 1 [file Table1.docx]

**Water-absorbent beads-induced ileal obstruction in children: a retrospective case series and translational rat model study**

**Supplementary experimental methods**

**Table1. Scoring criteria for disease activity index**

| **Weight Loss** | **Score** | **Stool Characteristics** | **Score** | **Rectal Bleeding** | **Score** |
| --- | --- | --- | --- | --- | --- |
| <1% | 0 | Formed granular stools | 0 | No Blood | 0 |
| 1%～5% | 1 | Soft stools | 1 | Visible minor blood streaks | 2 |
| 5%～10% | 2 | Pasty stools | 2 | Frank hematochezia | 3 |
| 10%～15% | 3 | Watery stools | 3 | Severe hemorrhage + signs of anemia | 4 |
| >15% | 4 | Bloody diarrhea | 4 |  |  |

**Table 2. Histological scores for intestine were assigned according to the modified criteria**

| **intestine** | | | | | |
| --- | --- | --- | --- | --- | --- |
| Crypt Architecture | Score | Goblet Cell Depletion | Score | Edema | Score |
| None | 0 | Absent | 0 | Normal | 0 |
| 1/3 crypt destruction | 1 | <50% area reduction | 1 | Mild edema (submucosal thickness 0.2 mm) | 1 |
| 2/3 crypt destruction | 2 | ≥50% area reduction | 2 | Moderate edema (submucosal thickness 0.21–0.45 mm) | 2 |
| Full crypt loss/ulceration | 3 | Complete loss | 3 | Severe edema (submucosal thickness >0.46 mm) | 3 |

**Table 3. qPCR Primer List**

| Gene | Protein name | Forward 5' -3' | Reverse 5' -3' | PMID |
| --- | --- | --- | --- | --- |
| Gapdh | Gapdh | CCCTTCATTGACCTCAACTAC | CTTCTCCATGGTGTGGTGAAGAC | None |
| Tjp1 | ZO-1 | GCCGCTAAGAGCACAGCAA | GCCCTCCTTTTAACACATCAGA | - None |
| Ocln | occludin | GCTTATCTTGGGAGCCTGGACA | GTCATTGCTTGGTGCATAATGATTG | None |
| Tnf | TNF‑α | GGAGGGAGAACAGCAACTCC | GCCAGTGTATGAGAGGGACG | None |
| Il6 | IL‑6 | CCAGTTGCCTTCTTGGGACT | CTGGTCTGTTGTGGGTGGTA | None |
| Il1b | IL‑1β | TTCTTTGAGGCTGACAGACC | CGTCTTTCATCACACAGGAC | None |
| Cldn3 | claudin‑3 | CTGCTCAGGCCATCTACGAG | CCCCAGCAGGATGCCAATTA | None |
| Muc2 | MUC2 | GCAAGGACTGTGTTTGCCTG | AGCACTTGTCATCTGGGTCG | None |
| Il10 | IL‑10 | CATTCCATCCGGGGTGACAA | TGTTGTCCAGCTGGTCCTTC | None |

**Table 4. Ordinal behavioural pain scale for rats**

| **Score** | **Behavioural criteria (undisturbed observation in home cage, 5 min observation after 2 min acclimatization under dim red light)** |
| --- | --- |
| 0 | Normal spontaneous activity and posture; no hunching, arching or abdominal guarding. Exploratory and grooming behaviours are intact. |
| 1 | Slight reduction in spontaneous activity; occasional transient hunching. No abdominal muscle contraction or abdominal guarding; abdomen maintains normal contact with cage floor. |
| 2 | Obvious reduction of movement; frequent hunching or back arching. Mild abdominal muscle tension, without lifting the abdomen off the cage floor. |
| 3 | Marked inactivity; persistent back arching. Strong abdominal contraction and partial lifting of abdomen off the cage floor. |
| 4 | Immobility, sustained severe abdominal guarding and pelvic elevation. No exploratory or grooming behaviour observed. |
